# P-209. Evaluation of Hospitalized *Clostridioides difficile (C. difficile)* Polymerase Chain Reaction (PCR) Positive/Enzyme Immunoassay (EIA) Negative Patients

**DOI:** 10.1093/ofid/ofae631.413

**Published:** 2025-01-29

**Authors:** Nicole Harrington, Drashti Patel, Jennifer Kelly, Clint Borja

**Affiliations:** ChristianaCare, Newark, Delaware; ChristianaCare, Newark, Delaware; ChristianaCare, Newark, Delaware; ChristianaCare, Newark, Delaware

## Abstract

**Background:**

Differentiating true infection versus colonization is challenging in *C. difficile* PCR positive/EIA negative patients. Prior studies estimate 50% of these patients have true infection and warrant treatment. The aim of this study was to describe treatment practices for hospitalized *C. difficile* PCR positive/EIA negative patients at ChristianaCare.

**Figure:**
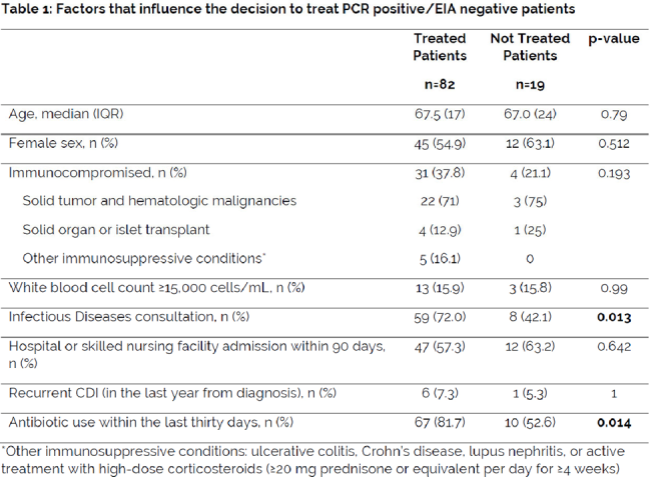

**Methods:**

A single health system, retrospective study was conducted between January to December 2022. PCR positive/EIA negative patients were categorized as treated or not treated for *C. difficile* infection (CDI). The primary outcome was the percentage of patients treated for CDI. Secondary outcomes that influence the decision to treat including age, sex, immunocompromised status, white blood cell (WBC) count, systemic antibiotic use, Infectious Diseases (ID) consultation, or recurrent CDI were evaluated. Hospital length of stay (LOS) and 30-day hospital readmission rates were also collected. ChristianaCare’s *C. difficile* scoring tool was used to categorize patients as low, medium, and high risk for true CDI.

**Figure:**
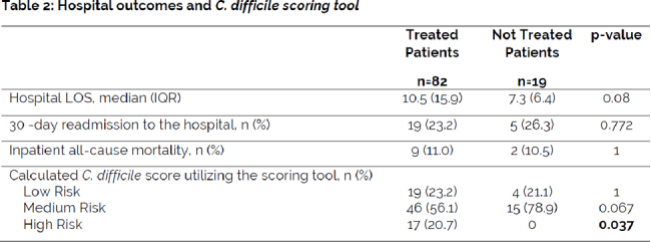

**Results:**

Of the 101 patients included in this study, 82 (81%) of PCR positive/EIA negative patients were treated for CDI (95% CI [0.73, 0.89]). Treated patients were more likely to have received antibiotics within the last 30 days (p=0.014) and have an ID consultation (p=0.013). There were no statistically significant differences between treated and not treated groups based on age, sex, immunocompromised status, elevated WBC, recurrent CDI, hospital LOS, 30-day hospital readmission, and inpatient all-cause mortality. No patients had real-time documentation of the *C. difficile* scoring tool score. All retrospective, manually calculated high risk patients utilizing the *C. difficile* scoring tool were treated (20.7%).

**Conclusion:**

We observed a higher percentage of treatment in patients who were *C. difficile* PCR positive and EIA negative compared to prior studies. Treated patients were more likely to have antibiotic use within the last 30 days and an ID consultation. Other risk factors commonly used to diagnose CDI did not impact treatment decisions. Opportunity to improve education using the ChristianaCare *C. difficile* scoring tool to assess CDI risk in patients and guide testing and treatment decisions is warranted.

**Disclosures:**

**All Authors**: No reported disclosures

